# Range of shoulder motion in patients with adhesive capsulitis; Intra-tester reproducibility is acceptable for group comparisons

**DOI:** 10.1186/1471-2474-9-49

**Published:** 2008-04-12

**Authors:** Einar Kristian Tveitå, Ole Marius Ekeberg, Niels Gunnar Juel, Erik Bautz-Holter

**Affiliations:** 1 Department of Physical Medicine and Rehabilitation, Ullevål University Hospital, University of Oslo, Norway

## Abstract

**Background:**

Measurements of range of motion play a key role in shoulder research. The purpose of this study is to investigate intra-observer reproducibility of measurements of active and passive range of motion in patients with adhesive capsulitis.

**Methods:**

The study was carried out in a population consisting of 32 patients with clinical signs of adhesive capsulitis. A specified measurement protocol was used, and range of motion in affected and non-affected shoulders was measured twice for each patient with a one-week interval.

**Results:**

For most of the investigated individual movements, test-retest differences in range of motion score of more than approximately 15° are not likely to occur as a result of measurement error only. Point-estimates for the intraclass correlation coefficient ranged from 0.61 to 0.93.

**Conclusion:**

Range of motion of patients with adhesive capsulitis can be measured with acceptable reproducibility in settings where groups are compared. Scores for individual patients should be interpreted with caution.

## Background

During the last few decades, increased focus has been placed on evidence-based medicine in the orthopedic disciplines. This development has made it essential to utilize instruments and methods that can detect clinically relevant changes. In shoulder research, measurements of range of motion (ROM) have been used as outcome measures in the vast majority of reported trials [1].

Shoulder ROM measurements can be divided into methods where passive or active movements are measured. In short, shoulder passive ROM is a measure of how far the observer can move the arm of the patient, while shoulder active ROM is a measure of how far the patient can move the arm himself.

The choice of an evaluative method is based on many considerations. What is crucial is high reproducibility, indicating little variation between measurements of the same quantity for the same individual. Measurement errors of shoulder ROM have been reported in several studies. Acceptable reproducibility of passive shoulder ROM measurements was reported in a study by Clarke et al. in 1974 [2], investigating healthy volunteers with a gravity-dependent goniometer. More recently, other researchers [3,4] have reported larger measurement errors when actual shoulder patients have been included. In one study [3], inter-observer differences between single observations of less than 20–25 degrees could not be distinguished from measurement error even with a well-adapted method. Such findings indicate that measurement errors for passive ROM are so large that important "true" changes may be misinterpreted. It has been argued that passive ROM is more difficult to measure reliably than active ROM [5]. Regarding shoulder measurements, however, there is limited evidence that measurement errors for active ROM are any smaller than for passive ROM.

Estimates of reproducibility of active ROM vary considerably. Some researchers investigating measurement errors for active ROM in shoulder patients have reported absolute measurement errors that are comparable to [6], or somewhat larger than [7,8], the results mentioned above [3,4] for PROM. Another group reported much larger measurement errors than this [9]. The conflicting results indicate that differences in procedures, study design or investigated population may have a large impact on reproducibility estimates. Gajdosik and Bohannon stated in a review article in 1987: "...results suggest a tremendous need for studies of the reliability of measuring ROM among the different patient types..." [5]. This statement may still be true today.

Adhesive capsulitis is a common cause of shoulder pain, estimated to affect 2% in the normal population [10]. It is characterized by a usually spontaneous onset of shoulder pain accompanied by progressive restriction in active and passive ROM of the glenohumeral joint. The objective of this study is to estimate intra-observer reproducibility of ROM measurements in affected and non-affected shoulders of such patients.

## Methods

As part of preparing for a randomized clinical trial (RCT) investigating patients with adhesive capsulitis, we developed a specific protocol for measuring range of motion in the shoulder. The reproducibility of these measurements was studied within the framework of the RCT project. The regional ethics committee granted ethical approval. The procedures followed protocol and complied with the Helsinki Declaration as revised in 1983 and current national ethical standards for such studies.

### Participants

Patients referred to the Ullevål University Hospital's Department of Physical Medicine and Rehabilitation in the time period August 2004 – June 2005 were considered for the study. Patients were assessed for eligibility according to the following criteria (same as for the RCT):

1. Limitation of passive movement in the glenohumeral joint compared with the unaffected side, more than 30 degrees for at least two of these three movements: forward flexion, abduction or external rotation. Patients with previous adhesive capsulitis in the opposite shoulder were accepted even if the differences between sides were somewhat smaller than 30 degrees. Patients were not eligible if they could not comply with passive range of motion measurement procedures due to e.g. excessive pain during measurements or huge difficulties in relaxing sufficiently to allow the investigator to make adequate recordings.

2. Pain in predominantly one shoulder lasting for more than 3 months, less than 2 years.

3. Willingness and ability to fill out shoulder self-report form.

Patients were included after informed consent unless they met any of the following criteria (same as for the RCT):

1. Diabetes mellitus (DM).

2. Trauma to the shoulder the last six months that required hospital care.

3. Serious mental illness.

4. Age under 18 or over 70.

5. Various contraindications to injections: allergy to injection material, blood coagulation disorders.

6. Patients with cancer and patients not expected to be able to follow treatment or follow-up protocol for practical or other reasons.

7. Patients currently taking corticosteroid tablets.

8. Reduction of glenohumeral range of motion for reasons other than "classic" adhesive capsulitis, e.g. X-ray signs of glenohumeral arthritis, dislocation or full-thickness rotator cuff tears with displacement of the humeral head.

Thirty-two patients were included. Nineteen participants (59%) were female, and mean age was 50 years (SD 6). Mean duration of the current episode was seven months (SD 4), and six of the patients had a history of frozen shoulder in the contralateral shoulder. Mean score of the Shoulder Pain and Disability Index (SPADI) [11,12] was 63 (0 is best, 100 is worst possible score). Mean restriction of glenohumeral passive ROM of the affected side compared to the non-affected side was approximately 60° for external rotation and approximately 45° for abduction, flexion and internal rotation.

### Measurements

**Figure 1:**
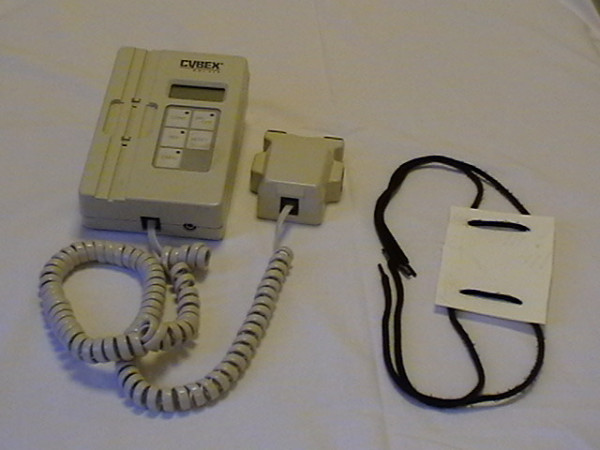
Inclinometer and plastic plate.

Several methods for shoulder ROM measurements have been proposed, using instruments that range from visual estimation [9,13-15] to still photography [9], goniometers [2,4,7,9,15-20] or advanced three-dimensional tracking systems [21]. We employed a gravity-dependent goniometer, also called an inclinometer (Cybex Electronic Digital Inclinometer, EDI 320 from Cybex Inc, Ronkonkoma, NY). Gravity-dependent goniometers have been used in recent clinical trials [22-25] investigating patients with adhesive capsulitis. They have also been tested in previous shoulder ROM reproducibility investigations [2,3,7,8,26-29]. These instruments are relatively inexpensive and easy to use. This particular instrument has a portable display and a hand-held unit. The display shows the change in position when the hand-held unit is rotated in the vertical plane. In order to produce a solid base for the hand-held unit for determining the different positions, we used a plastic plate with wraps that were easily attached to the patients' arms (Figure 1).

When the measurement protocol was worked out, we selected a strategy in which previously reported ways of measuring were employed to a maximum degree. Unfortunately, studies often do not give specific details on how measurements were made [1]. Furthermore, some methods reported in other studies were not suited for our study population. The measurement protocol is a mixture of previous recommendations, and to some extent, our own clinical experience. The protocol involved measuring the range of passive and active motion of four different movements of the shoulder.

#### 1. Abduction in the coronal plane

**Figure 2:**
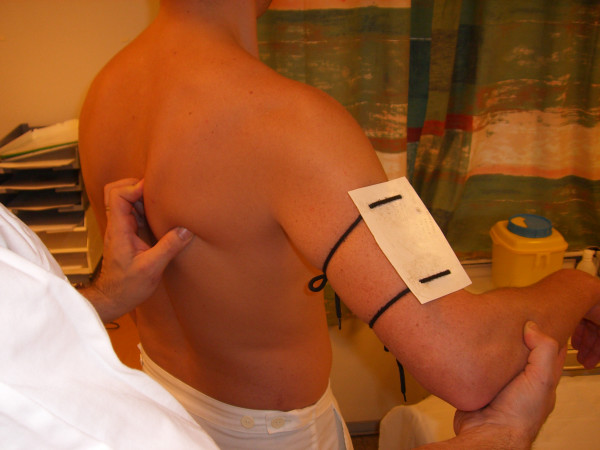
Passive abduction movement.

**Figure 3:**
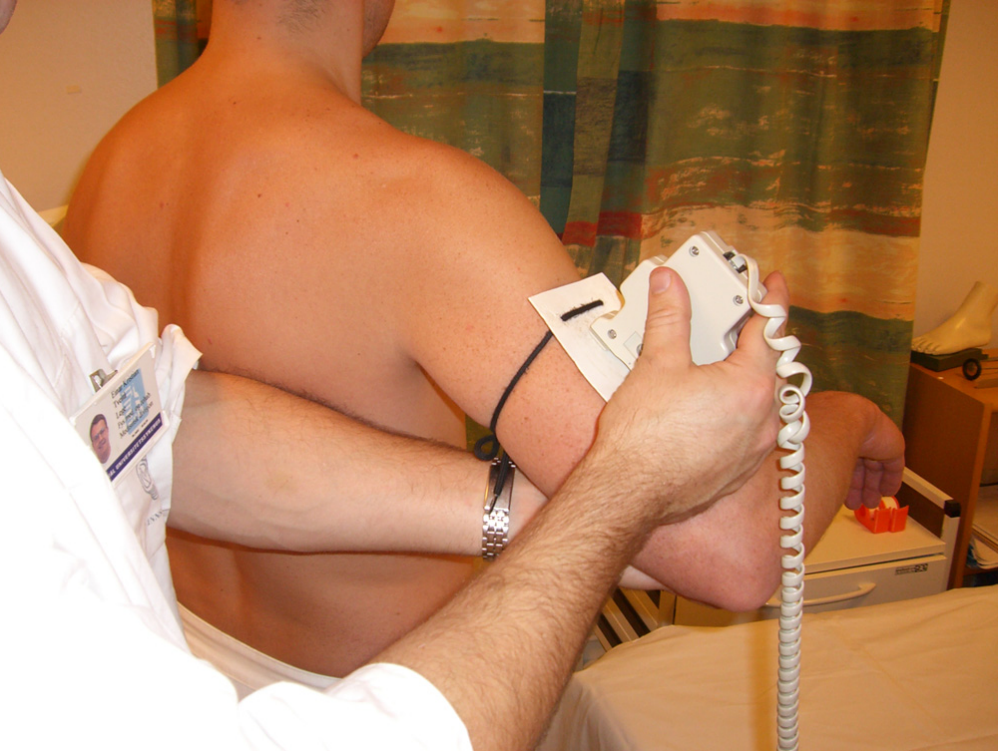
Passive abduction; arm stabilization and measurement of end-of-range point.

Passive abduction (P. ABD): The patient is standing. The plate is placed along the humeral shaft, laterally on the mid-section of the upper arm, and attached with the plate perpendicular to the abduction movement. The patient is instructed to relax. His elbow is held at 90° with the lower arm pointing forward under the entire movement. His upper arm is held parallel to the longitudinal axis of the body when determining the starting position. The physician stabilizes the patient's scapula with fingers holding the inferior angle. With the other arm, the physician then moves the arm carefully (Figure 2). The movement is stopped when resistance is met and the fingers holding the inferior angle of the scapula sense that the scapula is starting to move. At this end-point, the observer stabilizes the patient's arm and reads the inclinometer (Figure 3).

#### 2. Flexion in the sagittal plane

Passive flexion (P. FLE): The patient stands upright with his palms facing medially. The plate is placed along the biceps muscle, perpendicular to the flexion movement. The physician holds the arm parallel to the body's longitudinal axis, and instructs the patient to relax. The starting position is determined, and the inferior angle of scapula is stabilized as for P. ABD. The physician then moves the arm in the sagittal plane with minimum rotation of the arm. The end-point is determined and measured as for P. ABD.

#### 3. External rotation at 45 degrees of abduction

**Figure 4:**
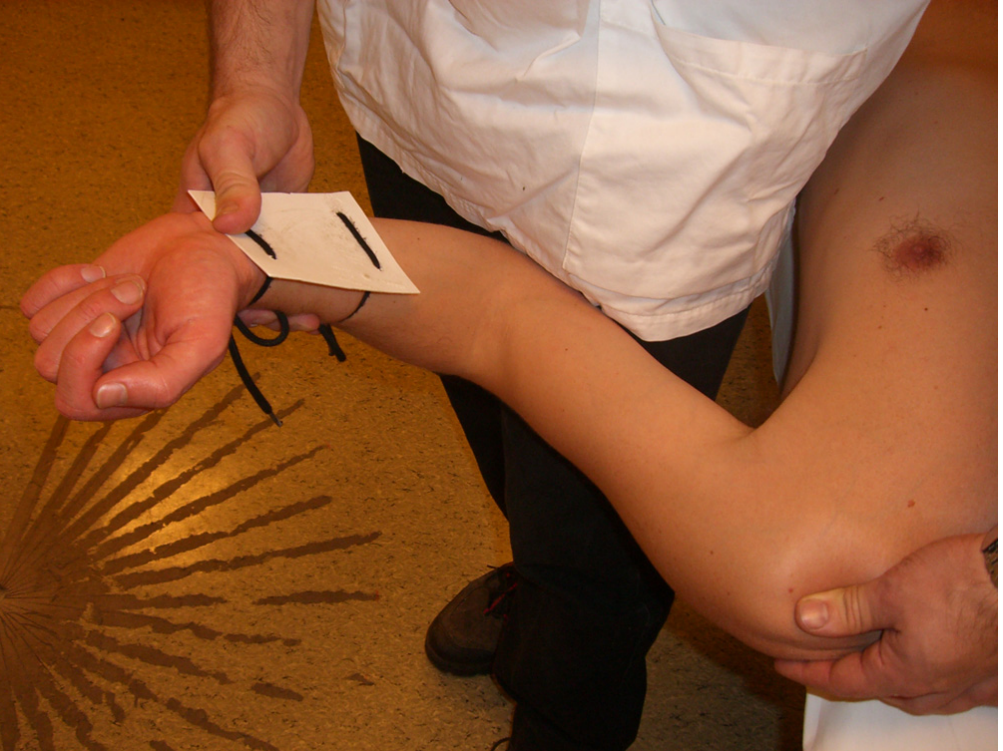
Passive external rotation movement.

**Figure 5:**
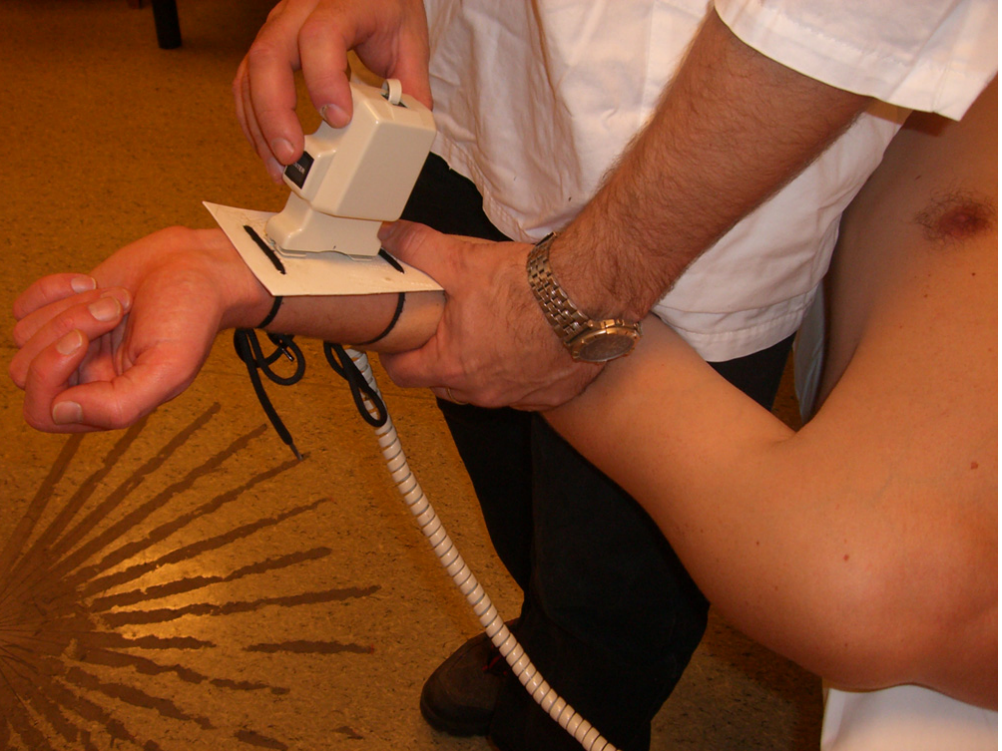
Passive external rotation; arm stabilization and measurement of end-of-range point.

Passive external rotation (P. EXT): The patient is lying down. The plate is placed along the distal, medial part of the ulnar and radial shafts. The patient's upper arm is held at about 45 degrees of abduction (allowing for necessary movement of the scapula in order to reach this position). The elbow is held at 90° with the lower arm vertical in the starting position. The physician stabilizes the acromion during the movement while the patient's arm is slowly rotated to the end-point (Figure 4). The plate is held perpendicular to the movement. The end-point is determined at the point where resistance to the movement is felt and before the acromion begins to move. The arm is stabilized and the inclinometer is read (Figure 5).

#### 4. Internal rotation at 45 degrees of abduction

Passive internal rotation (P. INT): The starting position is the same as for P. EXT, except that the plastic plate is attached to the lateral side of the lower arm. The arm is rotated internally. The rotation is stopped when resistance is met, and before the acromion begins to move. The arm is stabilized and the inclinometer read.

The corresponding measures of active ROM (A. ABD/A. FLE/A. EXT/A. INT) were made using similar patient starting positions as for the passive movements. The patient was instructed to move the arm as far as possible in the requested direction with minimal additional movement (change of wrist/elbow position, undue rotation, change in body posture and similar efforts). Scapular/clavicular movement was not restricted by the observer with these movements. When the inclinometer was read at the end-point, the plastic plate was held perpendicular to the movement. ROM was not measured during symmetrical movements.

Range of motion in both shoulders was measured twice for each patient with a one-week interval. This time interval was chosen because it seemed long enough for the observer to forget details concerning the first measurements, yet short enough to avoid any important change in "true" glenohumeral ROM for patients with this long-lasting [6,30] condition. The order of measurements was the same for both appointments. All measurements were made by the same observer. No patient started any new treatment in the one-week period.

### Statistical procedures

An overall measure of ROM was calculated numerically for the passive and active movements. It was defined as the sum of the four individual movements, and the measures were labeled "C. PROM" for the passive movements, and "C. AROM" for the active movements. Using single parameters to represent multiple movements may be unusual concerning shoulder ROM, but advantages with such parameters have been demonstrated when investigating motion in the cervical spine [31].

Reproducibility is reported in our study by both absolute and relative measurement error indices. Absolute measurement error (agreement) reflects the actual difference between observations. It is important when one wants to detect changes in health status over time [32]. Absolute measurement errors are in this study reported by the "within-subject standard deviation" (sw) derived from a one-way analysis of variance (ANOVA) as suggested by Bland and Altman [33]. We also report the "smallest detectable difference" (SDD). SDD, also known as repeatability, is defined as √2 × 1.96 sw = 2.77 sw [33]. The difference between two measurements for the same subject is expected to be less than the SDD for 95% of pairs of observations. Using a similar approach, we also report corresponding figures when levels of 90%, 80% and 50% are used.

The calculation of a common standard deviation for the measurements is based on the absence of heteroscedasticity [34]. Heteroscedasticity refers to a situation where measurement errors are dependent on the size of the various readings. We investigated the relationship between observed week-to-week differences and ROM means for each patient by using plots as proposed by Bland and Altman [33].

Relative measurement error is reported by the intraclass correlation coefficient (ICC). ICC is the correlation between one measurement on a target and another measurement obtained on that target [35]. ICC was computed using a one-way ANOVA model (single measures). Its values can theoretically range from 0 to 1. A high ICC indicates that the within-patient differences between the two measurements are small compared with the between-patient differences for this movement. Previous researchers have suggested that reliability above 0.70 is acceptable for group comparisons [3]. All statistical analyses were carried out using the software package SPSS 13.0 for Windows^® ^(SPSS, Chicago, IL, USA).

## Results

Results for reproducibility are given in Table 1 for PROM and in Table 2 for AROM. Observation 2 tended to display slightly lower scores than observation 1. According to paired t-tests, the difference was significant for P. ABD and A. ABD for the non-affected shoulder. In our analyses, we used a one-way ANOVA model to estimate absolute and relative reliability, and a possible and unexpected bias (approximately 1°–2°) was not corrected for by this method. However, we also calculated the indices using a two-way model (data not shown), where a possible session effect was included. This resulted in estimates that were quite similar to the ones obtained by the one-way model.

**Table 1:** PROM reproducibility. SD = between-subject standard deviation, CI = confidence interval, sw = within-subject standard deviation, SDD = smallest detectable difference, ICC = intraclass correlation coefficient.

**Movement**	**Side**	**Obs. 1**	**Obs. 2**	**Obs. 2-1**	**sw**	**SDD for various probabilities**				**ICC**
		Mean (SD)	Mean (SD)	Mean (95% CI)		95%	90%	80%	50%	(95% CI)

**P. ABD**	Affected	27° (9)	28° (9)	1° (-2 to 3)	5	14°	12°	9°	5°	0.72 (0.50–0.85)
	Non-aff.	73° (15)	70° (14)	-3° (-5 to -1)	4	12°	10°	8°	4°	0.89 (0.79–0.94)
**P. FLE**	Affected	40° (13)	41° (12)	1° (-2 to 4)	6	17°	15°	11°	6°	0.76 (0.57–0.88)
	Non-aff.	87° (12)	84° (11)	-2° (-6 to 2)	7	20°	17°	13°	7°	0.61 (0.34–0.79)
**P. INT**	Affected	31° (11)	29° (11)	-1° (-4 to 1)	5	14°	12°	9°	5°	0.81 (0.65–0.90)
	Non-aff.	73° (14)	73° (12)	0° (-3 to 2)	5	13°	11°	9°	4°	0.88 (0.76–0.94)
**P. EXT**	Affected	12° (16)	12° (15)	0° (-3 to 2)	5	13°	11°	9°	5°	0.91 (0.82–0.95)
	Non-aff.	71° (15)	70° (15)	-1° (-4 to 2)	6	15°	13°	10°	5°	0.86 (0.74–0.93)
**C. PROM**	Affected	109° (38)	110° (36)	0° (-7 to 7)	13	37°	32°	25°	13°	0.87 (0.76–0.94)
	Non-aff.	304° (39)	297° (40)	-6° (-12 to 0)	11	31°	27°	21°	11°	0.91 (0.82–0.95)

**Table 2:** AROM reproducibility. SD = between-subject standard deviation, CI = confidence interval, sw = within-subject standard deviation, SDD = smallest detectable difference, ICC = intraclass correlation coefficient.

**Movement**	**Side**	**Obs. 1**	**Obs. 2**	**Obs. 2-1**	**sw**	**SDD for various probabilities**				**ICC**
		Mean (SD)	Mean (SD)	Mean (95% CI)		95%	90%	80%	50%	(95% CI)

**A. ABD**	Affected	55 (21)	53 (21)	-2 (-5 to 1)	6	15	13	10	5	0.93 (0.86–0.96)
	Non-aff.	145 (13)	140 (16)	-5 (-10 to -1)	9	24	20	16	8	0.61 (0.34–0.79)
**A. FLE**	Affected	87 (24)	86 (23)	-1 (-6 to 4)	10	28	24	18	10	0.83 (0.67–0.91)
	Non-aff.	161 (10)	160 (10)	-2 (-4 to 1)	5	14	12	9	5	0.75 (0.55–0.87)
**A. INT**	Affected	45 (16)	40 (15)	-5 (-8 to 1)	7	18	15	12	6	0.77 (0.59–0.88)
	Non-aff.	90 (11)	90 (9)	-1 (-3 to 2)	6	16	14	11	6	0.69 (0.45–0.83)
**A. EXT**	Affected	18 (16)	16 (16)	-2 (-4 to 1)	5	13	11	9	5	0.91 (0.82–0.95)
	Non-aff.	80 (15)	80 (14)	0 (-3 to 3)	6	16	13	10	5	0.85 (0.71–0.92)
**C. AROM**	Affected	205 (57)	196 (60)	-9 (-19 to 0)	19	52	44	34	18	0.89 (0.79–0.94)
	Non-aff.	477 (31)	470 (36)	-7 (-15 to 1)	16	44	37	29	15	0.77 (0.58–0.88)

For PROM, estimated SDDs range from 12° to 20° for the individual movements and from 31° to 37° for the combined movement (C. PROM). For approximately 50% of pairs of PROM observations, test-retest difference was 5° or below (individual movements). SDD estimates for AROM ranged from 13° to 28° for the individual movements and from 44° to 52° for the combined movement (C. AROM). Estimated ICCs were in the area of 0.60–0.90 for both active and passive movements.

**Figure 6:**
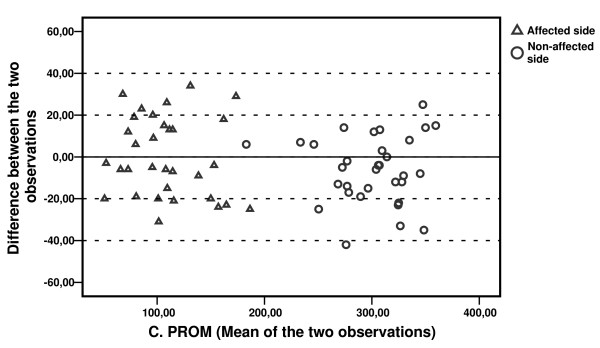
Mean C. PROM value of both observations plotted against the difference between observations for each patient

**Figure 7:**
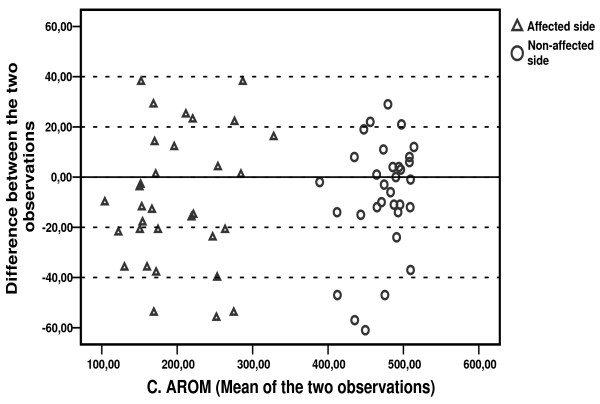
Mean C. AROM value of both observations plotted against the difference between observations for each patient

Figures 6 and 7 show the C. PROM and C. AROM difference between the two observations, plotted against the corresponding mean value of the two observations for each patient. Results for the affected and the non-affected side are presented within the same diagrams. Reproducibility seems to be independent of the magnitude of the score for the affected as well as the non-affected shoulder.

## Discussion

We have investigated the intra-observer reproducibility of measurements obtained with a standardized protocol for measuring range of motion in patients with adhesive capsulitis. Thirty-two patients were included in the study. This low number means that figures presented are only "rough" estimates of the reproducibility that can be expected in similar settings.

We observed a tendency for the scores of the second observation to be lower than those of the first observation. We have no good explanation for this possible bias, but would like to point out that any "true" within-patient changes in ROM during the one-week interval will lead to over-estimation of measurement error in a study of this type.

Even though we made a great effort to determine starting points and end-points consistently, this was probably a key source of error. The determination of these points involved deciding when the adequate patient/arm position was reached, the stabilization of arm position, placement of the inclinometer, and finally reading the display. These actions are all likely to add variability to measurements.

When measuring PROM, we stabilized scapula during movements, and used initiation of scapular movement to help determine end-points of glenohumeral motion. This procedure was not problem-free, and especially for flexion, it was sometimes difficult to determine when the scapula started to move. For all movements, patient muscle use could interfere with the passive movements, making representative scores difficult to obtain. Although we sometimes experienced problems with the technique, we share the view that it is essential to restrict scapulothoracic motion in order to more accurately reflect the range of motion of the glenohumeral joint [2,26,36]. There are also indications that stabilizing the scapula may produce more reliable results for some movements [16]. Furthermore, strict control of the scapula may be necessary in order to determine end-points of PROM with a method that does not cause excessive pain for the patient. The alternative might have been to determine end-point as the "point of first pain" [27] or as the point where pain makes further movement intolerable [14]. However, it is problematic to use pain as an end-point indicator in patients who experience pain more or less constantly, as many of the patients in our study did. It is our view that this would also affect validity when interpreting PROM values. When reported pain is used to indicate end-of-range, would we be measuring changes in the connective tissues surrounding the joint, or changes in patient pain status? Few clinical studies of patients with adhesive capsulitis have indicated how PROM end-of-range was determined. It will be advantageous if future researchers are more specific when reporting these procedures.

Testing positions are, of course, important when measuring ROM, and reproducibility is probably affected by the choice of testing positions [20]. In this study, we measured shoulder rotation from a 45° abducted position. It would have been more practical to measure rotation at 90° abduction, using positions described by Clarke [2] and others [16,17,26,27,29]. However, the rigid shoulders of our study population made it necessary to use a narrower angle.

Test positions and poor fixation of scapular movements may result in compensatory movements and thereby increased variation of measurements, in particular for active movements. Patients use various techniques to maximize their performance, some of which may be problematic to correct. Reliable (and valid!) measurements of these movements probably require very strict instructions to the patients. In many settings, however, there may be problems with the application of such methods.

We used a plastic plate in order to reduce "wobble" when applying the inclinometer. When experimenting without such a plate, we noted larger differences between subsequent measurements. We suspect that this method could be refined further, perhaps by using a longer plate, or stiffer material, or by improving plate attachment to the arm. Alternatively, attachment of the inclinometer itself to the arm during the entire movement, in a method similar to Clarke's [2,29], may be superior to the method we used.

For passive movements, smallest detectable difference (on the 95% level) was estimated to be approximately 15° for the individual movements. In a clinical setting, this indicates that differences between two single observations exceeding 15° can be trusted to represent a ''true'' difference. For some movements of active ROM, SDD (95%) was about 25°. Relative reproducibility estimates (ICC) in our study were 0.61–0.93 for the various movements. In general, reproducibility seems satisfactory for group comparisons, but may be insufficient for individual comparisons in many settings. However, confidence intervals are wide and indicate that larger studies are necessary for definite statements to be made. Riddle et al., investigating shoulder patients [19], reported intra-observer correlation of 0.87–0.99, but absolute measurement errors were not given. This makes comparisons problematic [34]. With the exception of this single study, measurement errors (absolute or relative) reported in comparable studies investigating intra-observer reproducibility in shoulder patient populations [4,7,9,27,28,37] are not smaller than the ones reported in our study of patients with adhesive capsulitis.

There was a tendency that SDD for AROM was larger than for PROM in this study. This may not be due to larger "measurement errors", strictly interpreted. It may be due to the patients' *exhibiting *truly more variable AROM than PROM. When measuring AROM, patients were asked to move their arm as far as possible in the requested direction, allowing for necessary scapular movement. The largest measurement errors in the study were noted for movements of active abduction and flexion, both of which are movements involving potentially large contributions by other joints. Furthermore, active ROM depends not only on the joints, but also very much on the overall function of the neuromuscular system. In addition to pain [14], AROM measurements may be seriously affected by motivation, learning effects or fear avoidance, all very fluctuating parameters.

One must consider that the patients in our study were selected to some degree. Because we wanted to study patients with adhesive capsulitis, we only included patients whose passive ROM could be adequately observed. There are undoubtedly some patients with painful shoulders whose ROM can only with difficulty be reliably and validly measured. This may be associated with pain [14], but the relationship is unclear [3]. One might speculate that other factors such as muscle tone, motivation or fear avoidance could play an important role. If this situation is suspected when ROM measurements must be made in patients, the use of anesthesia might be considered. Studying reproducibility under such conditions was beyond the scope of this project.

In this study, we employed a measure of "combined" total ROM. The corresponding SDD for PROM was 31° – 37°, meaning that differences larger than 8°–9° *on average *for four single movements are probably not due to measurement error. For combined total AROM, SDD was 44° – 52°, with corresponding figures of 11° – 13°. Average random error for each movement is reduced when several movements are combined like this [38]. Future researchers may want to investigate whether "combined" shoulder ROM is a useful parameter in various clinical settings.

## Conclusion

The present study of shoulder ROM measurements in patients with adhesive capsulitis indicates that reproducibility is acceptable for group comparisons, while scores for individual patients need to be interpreted with caution. We advise future researchers to carefully consider measurement protocols and study designs when investigating range of motion in patients with adhesive capsulitis.

## Competing interests

The author(s) declare that they have no competing interests.

## Authors' contributions

All authors contributed to study design. EKT recruited the patients, measured range of motion, performed the statistical analysis and drafted the manuscript. OME, NGJ and EBH helped to draft the manuscript. All authors read and approved the final manuscript.

## Pre-publication history

The pre-publication history for this paper can be accessed here:


